# Brain Fogginess and SIBO: A Link or Just a Mirage?

**DOI:** 10.1038/s41424-018-0055-y

**Published:** 2018-09-20

**Authors:** Sanjeev Sachdeva, A. S. Puri, Ajay Kumar, Ashok Dalal, Amol Sonyabapu Dahale

**Affiliations:** Department of Gastroenterology, GB Pant Hospital, New Delhi, 110002 India

We read the article by Rao et al.^1^, entitled “Brain fogginess, gas and bloating: a link between SIBO, probiotics and metabolic acidosis” with great interest. The authors deserve compliments for this thought-provoking study. This study has elegantly described a syndrome of brain fogginess (BF), gas, and bloating, possibly related to probiotic use, small intestinal bacterial overgrowth (SIBO), and d-lactic acidosis (DLA) in a cohort without short bowel. We have few pertinent comments regarding this study, which has addressed common functional gastrointestinal (FGI) symptoms of gas and bloating.

First, despite vast published literature on FGI disorders (FGIDs), the occurrence of concomitant BF has not been described previously in relation to FGIDs^2^. Also, BF is not reported to be among symptoms attributed to SIBO^3^. On the contrary, BF may be associated with certain syndromes like chronic fatigue syndrome^4^, fibromyalgia^5^, postural tachycardia syndrome^6^, and it may be triggered by a plethora of factors like lack of sleep, several drugs or toxins, alcohol or other addictions, metabolic or hormonal factors; and neurological conditions like Parkinsonism, dementia, ischemic brain disease and concussion injury^7^. Thus to attribute BF as a center-point of this study to SIBO needs a rethink and a meticulous assessment of all these entities needs to be undertaken before accepting this “link” as conclusive.

Second, diagnosis of BF in this study was quite subjective. It would have been desirable to have (a) objective tests of brain function like evoked potentials, EEG, (b) tests for minimal hepatic encephalopathy, (c) carotid Doppler and brain vascular and structural imaging like CT or MRI to rule out organic causes of BF.

Third, it is surprising to note that in patients with BF, duodenal aspirate culture were positive for SIBO in only 14/30 (46.7%), and positivity rate of glucose breath test (GBT) was merely 36.7%. This, in addition to the observation that reproducibility of BF on breath test was only 33% places a question mark on the proposed link between BF and SIBO.

Fourth, it is intriguing to note that only one out of 30 (3.3%) subjects with BF had elevated baseline urinary d-lactic acid level and only 9/30 (30%) had elevated peak serum l-lactic acid level. This places the proposed relation of brain fog to lactic acidosis in a quandary.

Fifth, frequency of lactic acidosis in those with BF and SIBO (by culture) was 57.1% as against 100% in BF with no SIBO (by culture). Also, a high proportion (68.4%) of patients with negative breath test for SIBO had lactic acidosis. Moreover, only 8/23 (34.8%) patients with d-lactic acidosis had culture positive SIBO. These observations make uncertain the proposed relation of SIBO to lactic acidosis. Lack of reproducibility of brain fog on breath testing in 10/30 (33.3%) subjects challenges the proposed link between SIBO and BF.

Lastly, mere assessment of symptoms but lack of post-treatment GBT or culture for SIBO to confirm success of therapy is a major limitation of this otherwise fascinating study.
